# Deep Learning for Anticancer Drug Discovery Targeting Non-Apoptotic Regulated Cell Death Mechanisms

**DOI:** 10.3390/ph19060851

**Published:** 2026-05-29

**Authors:** Mengwan Jiang, Jinlun Mu, Shuoye Yang, Peng Li

**Affiliations:** 1 School of Artificial Intelligence and Big Data, Henan University of Technology, Zhengzhou 450001, China; jiangmw@haut.edu.cn; 2 College of Information Science and Engineering, Henan University of Technology, Zhengzhou 450001, China; m17203990851@163.com; 3 School of Biological Engineering, Henan University of Technology, Zhengzhou 450001, China; yanshuoyecpu@163.com; 4 Institute for Complexity Science, Henan University of Technology, Zhengzhou 450001, China

**Keywords:** deep learning, anticancer drug discovery, regulated cell death mechanisms

## Abstract

Targeting non-apoptotic regulated cell death (RCD) modalities, such as ferroptosis and cuproptosis, offers a new avenue for overcoming resistance to conventional antitumor therapies, while deep learning provides a powerful tool for discovering bioactive molecules from multi-source data. This review delineates the core methodologies and application advances of deep learning in this domain, covering end-to-end molecular representations, multimodal fusion strategies, dataset partitioning criteria, and deep learning frameworks, thereby establishing a preliminary technical framework tailored to the study of non-apoptotic RCD mechanisms. Subsequently, the applications of deep learning in non-apoptotic RCD are discussed along three dimensions: direct applications, adjacent applications, and speculative outlooks. Future directions should focus on constructing high-quality annotated databases capable of distinguishing multiple cell death modalities and establishing standardized blind test benchmarks, developing explainable AI methods, designing mechanism-oriented few-shot learning algorithms, and building dynamic context-aware models. Advances along these directions may help propel the application of deep learning in drug discovery targeting non-apoptotic RCD mechanisms, from computational prediction toward experimental validation and translational research.

## 1. Introduction

Cancer, a major global public health challenge, has placed the innovation of therapeutic strategies at the core of biological research. While conventional chemotherapy and radiotherapy kill tumor cells, they are frequently associated with severe toxic side effects and drug resistance, prompting the continuous search for more precise and efficacious therapeutic approaches. Among these, discovering antitumor lead compounds from natural products is a primary avenue in antitumor drug discovery, owing to their structural diversity and abundant biological activities [1].

However, traditional drug discovery faces challenges such as cumbersome compound isolation and identification processes, low-throughput activity screening, and unclear mechanisms of action due to the vast amount of data. In recent years, a breakthrough advance in oncology research has been the revelation that tumor cells possess multiple cell death mechanisms beyond classical apoptosis, such as ferroptosis, cuproptosis, necroptosis, and pyroptosis. Their execution is independent of, or not limited to, the classical caspase cascade, but is instead driven and defined by a series of newly identified specific molecules (e.g., MLKL, Gasdermin, GPX4, FDX1), representing non-apoptotic RCD mechanisms [2,3,4]. These mechanisms provide new targets to circumvent traditional drug development but also impose higher demands on the precision and complexity of drug screening.

Against this backdrop, deep learning technology is empowering the field of drug discovery. Deep learning is a subfield of artificial intelligence. As defined by LeCun et al., it is a representation learning method that enables computational models composed of multiple processing layers to learn representations of data with multiple levels of abstraction [5]. It is capable of automatically extracting complex nonlinear patterns from massive multi-omics data, chemical structure information, and bioactivity data, thereby constructing an efficient predictive bridge from molecular structure to biological activity [6]. As these non-apoptotic RCD mechanisms exhibit fundamental differences in their biochemical execution machinery, distinct approaches must be adapted for different mechanisms.

In recent years, ferroptosis, cuproptosis, pyroptosis, and necroptosis have emerged as the four most representative forms of non-apoptotic RCD. Owing to their relatively well-characterized regulatory mechanisms, clearly defined key molecular events, and the continuously unraveled central roles they play in the onset and progression of major diseases, they are becoming a focal point of biomedical research, driven by deep learning models. This review primarily elaborates on the application of deep learning technologies under the four paradigmatic non-apoptotic RCD mechanisms: ferroptosis, cuproptosis, pyroptosis, and necroptosis.

This review aims to provide a structured narrative review of how deep learning is being applied to antitumor drug discovery targeting non-apoptotic RCD mechanisms. We conducted literature searches from the past five years using keywords such as “cell death mechanisms” and “deep learning,” supplemented with additional keywords tailored to specific section content. The inclusion criteria and ordering of studies were based on research impact, citation counts, and journal influence. The retrieved literature was first used to outline the deep learning methods and technical frameworks applicable to this field. Subsequently, we will detail, in sequence, the applications of deep learning in identifying compounds with cell death-inducing activity and drug-likeness, as well as in mining the non-apoptotic regulated cell death-inducing effects of known drugs. Finally, we will discuss the challenges and limitations facing current research and provide an outlook on future development directions.

## 2. Deep Learning Methods and Techniques for Non-Apoptotic RCD Mechanisms

Drug discovery is evolving from traditional experimentation towards intelligent prediction. Particularly in the field of cell death mechanisms, the complex biological networks and unique regulatory features make it challenging to systematically discover key targets and potent inducing compounds using traditional methods. Deep learning, at the forefront of AI technology, provides a powerful tool to overcome this bottleneck, leveraging its strengths in pattern recognition, automatic feature extraction, and complex relationship modeling. This section will delineate the core deep learning methods, key technologies, and practical pathways for investigating non-apoptotic RCD mechanisms.

### 2.1. Methods Adapted for Investigating Non-Apoptotic RCD Mechanisms

To better understand the application of deep learning in antitumor drug discovery, we first outline the widely adopted core algorithms in related research. Deep learning, a branch of machine learning, utilizes multi-layered neural networks to construct intelligent systems, with models categorizable by their output and architecture.

**Figure 1:**
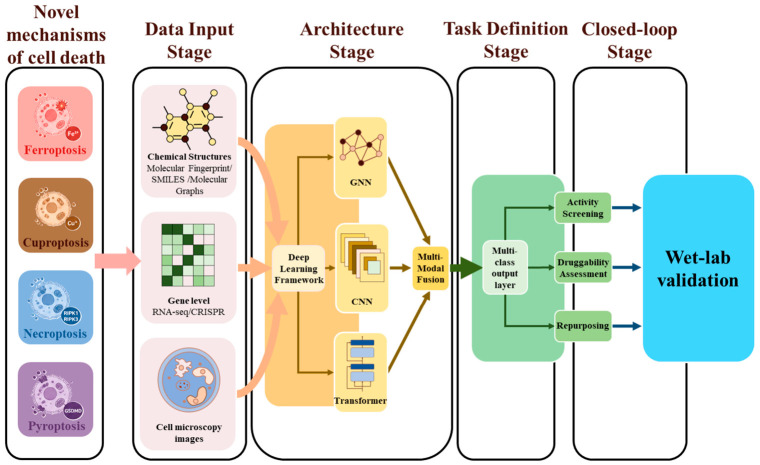
Basic workflow for constructing a deep learning framework. We divided the construction of a deep learning task under a non-apoptotic RCD mechanism into five components: the selection of the non-apoptotic RCD mechanism, the biological mechanism of this cell death, input data type, model architecture, and output task and validation strategy, with the biological mechanism summarized in Table 1.

A robust deep learning workflow typically involves these key steps: (1) defining the biological problem and translating it into a computable task; (2) collecting and preparing multi-source data; (3) performing feature engineering or employing end-to-end feature learning; (4) splitting data into training, validation, and test sets; (5) selecting or designing an appropriate network architecture; (6) training the model and evaluating its performance through cross-validation; and (7) applying the final model to the test set and iteratively optimizing it. Building upon this general workflow, deep learning methods must be further adapted to the following key aspects specific to the biological context of non-apoptotic RCD mechanisms. Figure 1 illustrates the fundamental workflow involved in constructing a deep learning architecture for investigating non-apoptotic RCD mechanisms.

1. Task Definition Granularity

Traditional drug screening often uses cell viability as an endpoint, merely indicating whether a compound is cytotoxic. Cell death research requires models to distinguish the type of death. Consequently, the task must upgrade from binary classification to multi-label or multi-class classification problems. This upgrade directly leads to a sharp increase in sensitivity to label noise, because distinguishing pyroptosis from necroptosis relies on biomarkers rather than simple cell viability, potentially even introducing ranking-based learning to assess the activation intensity of different death pathways. In training label construction, multi-modal experimental data, such as ferroptosis and necroptosis markers, can be integrated to form a composite supervisory signal. The task definition must be consistent with the biological detection criteria for the death mechanism. As shown in Table 1, different non-apoptotic RCD mechanisms have distinct detection methods and impose different requirements on models.

2. Multimodal Data Fusion

Non-apoptotic RCD mechanisms are subject to multi-level regulation; a single data source is insufficient to fully characterize them. Hence, advanced models frequently employ multimodal fusion strategies. At the molecular level, drug chemical structures are encoded using SMILES strings or molecular graphs. At the gene level, RNA-seq, CRISPR screening data, or pathway enrichment scores are integrated as contextual features. At the phenotypic level, high-content imaging or bright-field microscopy images are input, with morphological features extracted by CNNs.

Notably, dataset splitting strategies under non-apoptotic RCD mechanisms require careful consideration. Random splitting of molecules into training and test sets is common, but it may lead to an overestimation of the model’s true generalization ability. The splitting should be tailored to the specific data characteristics of the non-apoptotic RCD mechanism under study. For instance, Guo et al. compared four splitting strategies (random, scaffold, Butina clustering, and UMAP clustering) and found that UMAP-based splits provided a more challenging and realistic benchmark for model evaluation, whereas scaffold and random splits both overestimated model performance [7]. Ektefaie et al., using the SPECTRA framework to systematically evaluate the generalization of deep learning models for chemical property prediction, found that even with emerging methods such as UMAP splits, model performance might still be optimistically estimated [8]. Witman et al. highlighted that data leakage caused by random splits can severely overestimate the out-of-distribution (OOD) generalization ability of models, and proposed MatFold, a systematic cross-validation framework based on structure, composition, and chemical system [9].

If highly similar analogues exist in both training and test sets, the model may only learn to interpolate local chemical patterns rather than genuinely extrapolating the essential pharmacophores and molecular mechanisms required for inducing cell death. More critically, the scarcity of active molecules against non-apoptotic RCD mechanisms renders models highly susceptible to overfitting to the few positive samples, generating spurious correlations. Aouichaoui et al. discovered that random or clustering-based splits could lead to certain key molecular functional groups being “missing” from the training set, forcing the model to fail to learn the core chemical features determining a property and instead rely on local similarity for “interpolation” [10]. To address this, they proposed a “minimum coverage” splitting strategy ensuring the training set encompasses all chemical substructures. An improper split, when key pharmacophores are sparse within a dataset, hinders the model from learning their defining mechanism, and this scarcity itself exacerbates the risk of overfitting spurious correlations.

3. Network Architecture Tailoring

Selecting or customizing appropriate architectures is necessary for different data types. Graph neural networks, convolutional neural networks, Transformers, and generative models represent the deep learning solutions applied to non-apoptotic RCD mechanism research. Furthermore, to tackle the scarcity of annotated data, self-supervised pre-training and transfer learning are widely adopted to enhance generalization performance under few-shot conditions. The specific deep learning frameworks and tools for studying non-apoptotic RCD mechanisms will be detailed in Section 2.3.

4. Closed-Loop Validation

The final model output must feed back into biological validation. The ideal workflow should form a closed loop of “prediction, screening, experimental confirmation, and model iteration.”

Currently, most studies only report model performance on a held-out test set, lacking independent external experimental validation as a standard. Although some works include experimental validation, it is often limited to case demonstrations of a few predicted hit compounds, without systematically reporting the success rate, false positive rate, and breadth of validation coverage on external datasets. This makes it difficult to objectively assess the practical utility of models in real drug discovery scenarios [11]. Gangwal et al. proposed the IMPACT framework, calling for rigorous benchmarking, blinded validation, and uncertainty quantification, elevating evaluation from “internal testing” to “independent validation” [12].

**Table 1:** Biological nature of non-apoptotic RCD mechanisms and their constraints on modeling. The table elucidates the biological principles underlying four non-apoptotic regulated mechanisms of cell death, thereby providing the necessary biological foundation for constructing a deep learning task.

Death Mechanism	Core Biochemical Events	Key Execution Molecules	Endpoint Detection Methods	Impact on Modeling
Ferroptosis [13]	Lipid peroxidation, iron-dependent ROS	GPX4 inactivation, ACSL4, LPCAT3	Lipid ROS probes (C11-BODIPY), MDA content, ferrostatin rescue	Ferroptosis is a dynamic, multi-stage process. Modeling necessitates time-series data to capture the critical transition from “antioxidant defense” to “uncontrolled lipid peroxidation.”
Cuproptosis [14]	Copper ion accumulation, DLAT oligomerization, Fe-S cluster protein degradation	FDX1, DLAT, LIAS	Copper ionophore dependence, FDX1 knockout rescue, DLAT oligomerization staining	Cuproptosis is a highly concentration- and target-specific process. Models must learn the nonlinear boundary between “normal copper metabolism” and the “copper lethality threshold.”
Necroptosis [15]	RIPK1/RIPK3/MLKL phosphorylation cascade, membrane pore formation	pMLKL, RIPK3	pMLKL detection, necrostatin-1 rescue, PI-positive but caspase-negative	Necroptosis shares signaling overlaps with apoptosis and pyroptosis. Models must distinguish this “mixed signal,” which imposes requirements for multi-task learning frameworks.
Pyroptosis [16,17]	Caspase-1/4/5/11 cleavage of Gasdermin D, membrane pore formation	GSDMD-NT, Caspase-1	GSDMD-NT release, IL-1β secretion, LDH release, caspase-1 inhibition	Pyroptosis is an explosive event; cell fate determination possesses a steep, switch-like characteristic, demanding high precision and real-time capability from model predictions.

### 2.2. Molecular Descriptors and Data Representing Non-Apoptotic RCD Induction Capacity

In applying deep learning models to predict and discover antitumor drugs, effectively representing molecular structure information is the primary critical step. Molecular “representations” or “descriptors” form the basis for model learning and directly affect the accuracy of predicting cell death induction capacity. This section outlines several major types of molecular descriptors and their manifestations in data. Although these descriptors are encodings of general chemical information, selecting appropriate, information-rich representation methods is crucial for the model’s success in capturing key structural features underlying the complex biological phenotype of cell death induction.

#### 2.2.1. Traditional Molecular Descriptors

In early drug discovery, identifying candidate molecules with desirable physicochemical features is a critical prerequisite for their successful development into effective drugs [18]. Molecular descriptors and fingerprints, as core tools in computational chemistry, aim to quantify these features of chemical entities and their biological targets, thereby providing numerical inputs for deep learning-based models.

Traditional molecular descriptors are numerical representations derived from theoretical calculations or experimental measurements of a molecule’s physical, chemical, or structural properties [19]. They encompass a wide range, from simple physicochemical properties (e.g., molecular weight, logP, number of hydrogen bond donors/acceptors) to more complex topological descriptors (e.g., indices encoding molecular connectivity). This category is also termed “expert-knowledge-based features,” relying on predefined rules and algorithms by domain experts to extract molecular information. They are characterized by computational efficiency, fixed feature dimensionality, and excellent performance on tasks with small datasets [20,21]. For ferroptosis, where the core mechanism is relatively clear, features defined by expert knowledge are a relatively good choice, as the specific process can be clearly represented. For cuproptosis, a newer concept with extremely scarce known active molecules, traditional molecular descriptors are also a relatively preferred option, avoiding issues such as overfitting due to data scarcity.

In comparison, molecular fingerprints are a more complex, specialized type of descriptor. They typically encode the presence or absence of specific substructures or paths within a molecule as a binary bit string [22]. For ferroptosis, molecular fingerprints are a sub-optimal choice because common fingerprints, while indirectly reflecting substructural patterns, are not specifically designed to encode reactivity features and may miss crucial information about redox-active groups.

These traditional descriptors play an indispensable role in deep learning-driven drug discovery, with applications spanning target molecule ranking and similarity-based compound searching, virtual screening of large chemical libraries, quantitative structure–activity relationship (QSAR) analysis, and ADMET prediction for lead compounds [23,24].

#### 2.2.2. End-to-End Molecular Representation

Although traditional descriptors are computationally efficient and possess clear chemical interpretability, their reliance on expert knowledge for design and selection introduces potential information loss and human bias [25]. To overcome these limitations, as comprehensively discussed by Chuang et al., new deep learning-based methods are being deployed to automatically extract features from raw data through end-to-end learning, thereby improving the performance of compound bioactivity prediction models [26]. Molecular end-to-end learning operates on input forms such as SMILES, SELFIES, and molecular graphs.

The Simplified Molecular Input Line Entry System (SMILES) is one of the most commonly used string representations, translating a molecular structure into a sequence of characters governed by specific syntax rules [27]. Its advantages lie in data abundance, compact storage, and the ability to leverage mature models from Natural Language Processing (e.g., RNNs, Transformers) for processing [28]. For example, the ChemBERTa model (ArXiv preprint) proposed by Chithrananda et al. is a Transformer-based chemical large language model pre-trained via self-supervised learning on massive molecular datasets [29]. It learns rich chemical semantic information, significantly boosting downstream task performance. However, SMILES has notable drawbacks: a single molecule can have multiple valid SMILES strings, leading to non-unique representations; its syntax structure is relatively fragile, where a single character error can yield an invalid molecule, posing challenges for model learning [30].

SELFIES (Self-Referencing Embedded Strings) emerged as an innovative alternative to SMILES [31]. The design principle of SELFIES ensures that every possible string corresponds to a valid molecular structure. This characteristic makes it tremendously potent for molecular generation tasks, fundamentally resolving the issue of generative models producing invalid chemical structures and greatly simplifying model training. Since its introduction, SELFIES has been rapidly adopted in various generative model frameworks and has demonstrated powerful capabilities in designing molecules with specific properties [32]. However, this representation suffers from poor human readability.

The molecular graph is the most natural and information-complete representation of a molecule [33] (ArXiv preprint). It represents atoms as nodes and chemical bonds as edges, directly constituting the graph structure of the molecule. Graph neural networks (GNNs) are the mainstream technique for processing such data. Through a message-passing mechanism, they aggregate information from neighboring atoms, automatically learning hierarchical features from local atomic chemical environments up to the whole-molecule level [34]. Molecular graph representation overcomes the limitations of string-based methods, naturally capturing molecular topology, and has become the best-performing and most widely adopted end-to-end learning method for molecular property prediction [35]. In recent years, GNN architectures have continuously evolved; for instance, the Attentive FP model by Xiong et al. introduced graph attention mechanisms, enabling the model to focus on atoms and chemical bonds that are more relevant to the task [36]. Additionally, models such as Graph Transformers, which combine the Transformer architecture with graph structures [37], further enhance the capability to capture long-range dependencies.

End-to-end representations, also known as learnable representations, utilize deep learning models to automatically learn features directly from a molecule’s raw representation. They are characterized by high adaptability and performance dependence on data scale [21,38,39,40]. For pyroptosis and necroptosis, end-to-end learning is theoretically attractive because the available data volume is large. Unlike ferroptosis, determining pyroptosis and necroptosis relies on specific biomarker detection, not generic cell viability assays. However, label noise in the data constitutes a major obstacle. The vast majority of current large-scale public cytotoxicity databases record only simple live/dead endpoints, lacking death subtype information. Consequently, if end-to-end models are trained directly on these large but label-contaminated datasets, what they learn is, to a large extent, the recognition of “pan-cytotoxicity,” and they may not specifically point towards pyroptosis or necroptosis.

#### 2.2.3. Multimodal Data Fusion

While the aforementioned traditional descriptors and end-to-end learning approaches represent molecular structure from distinct angles, they primarily rely on single-source chemical structure information. When predicting a compound’s cell death induction capacity, chemical structure alone might be insufficient to capture its complex biological effects. Although Stokes et al. demonstrated the value of chemical structure in predicting bacterial growth inhibition, the more complex phenotype of cell death likely necessitates integrating additional biological information (e.g., transcriptomic data or protein interaction networks) to enhance prediction accuracy [6]. To construct more accurate and robust predictive models, multimodal data fusion offers a powerful solution. As investigated by Zitnik et al., fusing multimodal data through graph convolutional networks allows for more comprehensive modeling of complex drug interactions, providing a methodological reference for integrating multi-source data in our research context [41].

The effectiveness of multimodal fusion strategies is highly dependent on the quality and annotation accuracy of the data itself, which constitutes the core bottleneck for applying deep learning to drug discovery targeting non-apoptotic RCD [42]. Currently, relevant public data resources exhibit a clearly hierarchical landscape (see Table 2). General compound databases provide vast amounts of chemical structure and basic activity information but generally lack fine-grained annotation of cell death types [43]. Multi-omics and drug perturbation databases offer model capability for biological context awareness; however, their endpoints are predominantly cell viability, making it difficult to distinguish specific death modes like ferroptosis or cuproptosis [44]. Phenotypic image repositories and knowledge graphs can supplement morphological evidence and prior pathway knowledge, yet specifically annotated data for non-apoptotic RCD mechanisms remain extremely scarce [45,46]. Notably, specialized disease-specific databases, represented by FerrDb, offer high-precision and experimentally validated annotations, providing gold-standard accurate data for model training.

In this context, the core of multimodal data fusion lies in combining molecular chemical structure information with relevant biological contextual information [47]. For the task of predicting cell death induction capacity, key fusion modalities include the following four. (1) Chemical structure modality serves as the base modality, employing traditional molecular descriptors, molecular fingerprints, or deep learning embedding vectors derived from end-to-end learning on SMILES, SELFIES, and molecular graphs [48]. (2) The bioactivity profile modality represents the preliminary activity data of a molecule across multiple targets or cell-based phenotypic assays. For instance, a compound’s inhibitory activity profile against a panel of kinases or GPCRs can serve as an indirect indicator of its potential mechanism of action and cell death pathway engagement [49]. (3) The genomics/transcriptomics context modality represents the mutational status or gene expression profiles of tumor cells [50]. Since the same compound may induce different forms of cell death in cell lines with distinct genetic backgrounds, integrating molecular information with cell line genomic features can significantly improve prediction accuracy and interpretability [51]. (4) the physicochemical and pharmacokinetic modality represents deeper ADMET properties that can be used as an independent modality to determine whether a molecule possesses the ability to reach its site of action and exert its function [52].

Fusion strategies primarily fall into three categories [53]:

1. Early fusion concatenates feature vectors from different modalities at the input level before feeding them into a single model. This method is simple but struggles with handling missing modalities and may ignore complex inter-modal relationships [53].
2. Late fusion trains independent prediction models for each modality and then integrates the final prediction results from each model. This is flexible but cannot capture the underlying correlations between modalities [54].
3. Intermediate fusion is widely considered an effective strategy balancing expressiveness and flexibility [32]. It involves first learning low-dimensional dense representations for each modality using dedicated encoders, and then integrating them within intermediate layers of the model through concatenation, attention mechanisms, or tensor fusion. This approach captures complex cross-modal interactions while maintaining model flexibility [55].

In existing modeling studies on ferroptosis and cuproptosis, mid-term fusion is a strategy that has been frequently adopted and has shown great potential, with its success being highly dependent on high-quality, paired multimodal data. The core features of ferroptosis and cuproptosis share some downstream effects; early fusion (simple concatenation) would ignore the differences in their unique metabolic pathways, while late fusion (independent modeling) would struggle to capture their synergistic lethal effects [56,57]. In contrast, for pyroptosis and necroptosis, which are often accompanied by dramatic cellular morphological changes, accurate differentiation relying solely on traditional single-molecule markers is difficult. Hence, high-resolution intermediate or late fusion based on morphology is typically employed [58] (bioRxiv preprint). The morphological changes in pyroptosis are highly complex and heterogeneous. Using a dedicated encoder to extract morphological features (intermediate fusion) or aggregating multimodal prediction scores (late fusion) can capture subtle biological differences more effectively than simply concatenating pixel values (early fusion). In the current interdisciplinary research on non-apoptotic RCD mechanisms, intermediate fusion (especially architectures based on attention mechanisms or Transformers) is a promising approach, balancing biological mechanistic interpretability and model flexibility [59]. Early fusion is too coarse to handle the unique, high-dimensional, sparse markers of each death mechanism; late fusion might miss the well-established metabolic crosstalk between ferroptosis and cuproptosis.

Through multimodal data fusion, model predictions evolve from single activity assessments based on compound structure to systematic efficacy predictions integrating multidimensional chemical, biological, and genomic information. As noted by Vamathevan et al., the successful application of deep learning in drug discovery hinges on the systematic integration and joint analysis of multi-source data, such as genomics, imaging, and chemical structures [60]. However, the degree of reliance on different fusion modalities varies intrinsically between death mechanisms, which directly determines the choice of fusion strategy during model design.

Based on current understanding, the chemical structure of iron may contain key pharmacophore information for inducing ferroptosis. This was validated in the ChemProbe model developed by Connell et al., which successfully predicted the cell-line-specific sensitivity of ferroptosis inducers ML162 and RSL-3 by conditionally embedding chemical structure features into the gene expression space [61]. For cuproptosis, because the key event is copper ion accumulation in specific organelles, the fusion of the physicochemical property modality with the mitochondrial gene expression background may become a necessary condition [62]. For necroptosis and pyroptosis, models must rely on the genomics context modality, particularly the expression status of key nodes in the RIPK1/RIPK3/MLKL or Caspase/GSDMD pathways, to make meaningful death type predictions [63,64]. This differentiated dependency on modalities dictates the selection of fusion strategies in model design.

Through this differentiated modality integration, models can learn complex rules, such as: “A natural product containing a specific electrophilic warhead, within a tumor cell possessing a particular metabolic reprogramming signature, is more inclined to induce the cell death mechanism of cuproptosis.” This capability allows for more precise identification of natural active molecules with potent cell death induction capacity during virtual screening and provides valuable hypotheses regarding their potential mechanisms of action [65].

**Table 2:** Key data resources for research on non-apoptotic RCD mechanisms. The table summarizes publicly available datasets spanning general compound libraries, multi-omics and drug perturbation resources, drug–target association databases, protein structure repositories, phenotypic imaging and knowledge graph platforms, and mechanism-specific databases. Detailed contents of the table are presented in Supplementary Table S1.

Resource Category	Resource Name	Core Data Types	Major Limitations	Annotation Content
General Compound Libraries	PubChem, ChEMBL, ZINC [66,67,68]	Chemical structures, basic bioactivity	Lack cell death type annotation; high data heterogeneity	Chemical structures and basal bioactivity
Multi-omics & Drug Perturbation Libraries	PRISM, DepMap, CCLE, GDSC, CTRP, CMap, LINCS [49,50,69,70,71,72,73]	Transcriptomes, genotypes, drug sensitivity data	Endpoints do not distinguish death modes (mostly cell viability)	Drug sensitivity and omics features
Phenotypic Images & Knowledge Graphs	Cell Painting, IDR, KEGG, Reactome (version 2011) [74,75,76,77]	High-content images, signaling pathway networks	Scarcity of disease-specifically annotated images; static pathways hard to reflect dynamic regulation	Morphological features and pathway information
Drug-Target Association Databases	DrugBank, BindingDB [78,79]	Drug-target, protein-ligand binding data	Not specialized for cell death information; requires integration with pathway databases or downstream validation	Drug-target binding and affinity parameters
Protein Structure Database	PDB [80]	Protein 3D structure	Static structures cannot capture dynamic cell death processes	Atomic-level 3D coordinates and active site information
Specialized Disease Databases	FerrDb [81]	Experimentally validated inducers/inhibitors, targets, mechanisms	Covers only a single death type; equivalent resources for other mechanisms are lacking	Experimentally validated death regulators with mechanism-classified annotation

### 2.3. Deep Learning Networks for Investigating Non-Apoptotic RCD Mechanisms

We begin with a brief introduction to several fundamental deep learning architectures that are most relevant in the drug discovery context (see Table 3). Deep learning models are typically classified by their network structure and task objectives, mainly including graph neural networks, convolutional neural networks, Autoencoders, and generative models. Different architectures are designed to handle specific data types and accomplish corresponding tasks, such as molecular property prediction and compound generation [82]. We also specified the applicable scenarios and challenges in non-apoptotic RCD mechanisms.

Graph neural networks have been increasingly employed to predict compound-induced cell death activities. Liu et al. utilized GNNs to predict synthetic lethal interactions in cancer, achieving a mean accuracy of 0.817 and demonstrating superiority over alternative architectures such as GAT and GTN [83]. Although this work was conducted in the context of cuproptosis, it should be noted that in settings where active compounds are scarce—as is the case for cuproptosis—the predictive performance of such models remains contingent on access to a sufficient volume of high-quality training data [84]. Convolutional neural networks have been applied to high-content screening images to classify cell death modalities based on morphological alterations; however, these models are prone to learning spurious correlations linked to experimental treatment conditions rather than the underlying death mechanisms and are therefore better suited as orthogonal validation tools [85,86]. Recurrent neural networks and Transformers have shown value in generating SMILES sequences for potentially active novel molecules. Li et al. proposed ConfBiXtCPI, a bidirectional cross-attention Transformer that captures molecular recognition patterns directly from sequence-level inputs, achieving state-of-the-art predictive accuracy across multiple benchmark datasets [87]. Nevertheless, existing large-scale cell viability datasets typically lack annotations that distinguish necroptosis from pyroptosis, and the resulting label noise substantially compromises the generalization capacity of Transformers in multi-task prediction settings [88]. Autoencoders and variational autoencoders are highly dependent on the breadth of chemical space covered by their training data; when trained solely on the limited set of known inducers of non-apoptotic RCD, their generative outputs remain confined to established chemotypes, hindering scaffold hopping and innovation [89]. Multimodal deep learning remains at the proof-of-concept stage. Ye et al. proposed FViM, a Vision Mamba-based architecture for predicting cell death pathways [90]. However, the most fundamental challenge in this direction is the acute scarcity of high-quality, paired multimodal datasets, which leads models to capture shallow statistical associations across modalities rather than deep causal mechanisms, producing biologically plausible yet experimentally unverifiable hypotheses [91,92] (ref. [92] is ArXiv preprint). Graph attention networks have shown promise in dissecting cell death signaling pathways and identifying regulatory targets. Wong et al. developed a GAT-based biological pathway model capable not only of predicting pathway dynamics but also of rediscovering all five gene–gene interactions within the canonical TP53–MDM2–MDM4 feedback loop directly from gene expression data, highlighting the strong potential of such models to generate novel biological hypotheses de novo [93]. Critically, however, the direct output of these models is target importance rather than compound activity, rendering their application to predicting cell death-inducing capacity an indirect inference whose reliability is fundamentally governed by the quality of the underlying network construction [94].

Next, we delve into a suite of cutting-edge deep learning tools that are reshaping the research landscape of non-apoptotic RCD mechanisms and discuss how they are being employed to distinguish and elucidate different forms of non-apoptotic RCD.

In the realm of protein structure prediction, AlphaFold 2, proposed by Jumper et al., is undoubtedly a milestone [95]. It utilizes a revolutionary attention mechanism to predict three-dimensional protein structures from amino acid sequences with high accuracy, but its core competency is predicting static structures. Cell death processes, however, involve dynamic conformational changes, such as protein complex assembly. Therefore, AlphaFold 3, proposed by Abramson et al., is particularly critical [96]. It significantly expands the prediction scope to include complexes of proteins, DNA, RNA, small molecules, and ions and can simulate co-folding processes, theoretically providing a powerful tool for studying processes such as copper ionophore-induced protein aggregation in cuproptosis. Similarly, RoseTTAFold, based on a three-track network architecture proposed by Krishna et al., can even be used for the de novo design of functional death-inducing or death-inhibiting peptides [97]. New models in this direction are demonstrating unique advantages; for example, Boltz-1 and Boltz-2, proposed by Wohlwend et al., improve the accuracy of protein–ligand binding interface prediction by explicitly modeling water mediation effects [98,99] (ref. [99] is a bioRxiv preprint). Chai-1, proposed by the Chai Discovery team, is compatible with various post-translational modifications and non-natural amino acids [100] (bioRxiv preprint). Collectively, these tools are expanding the application boundaries of structure prediction in drug design and are being benchmarked against traditional docking methods, with results showing significant improvements in sampling efficiency and accuracy in certain scenarios. A key challenge, however, lies in distinguishing whether the high-affinity binding predicted by these models represents a true inhibitory signal or is merely a “hallucination” stemming from the model’s bias towards specific residue distributions.

It must be emphasized that static structure prediction alone cannot reveal selectivity in cell death pathways. Taking GPX4 as an example: AlphaFold can accurately predict the binding mode of GPX4 with its inhibitors (such as RSL3), but it cannot answer the critical biological question of whether covalent modification by the same inhibitor will lead to ferroptosis, necroptosis, or apoptosis in different cellular contexts. This is because the downstream death execution pathway following GPX4 inactivation depends on the cell’s metabolic background and compensatory signals. Therefore, structure prediction must be combined with pathway-level multi-omics models to translate target engagement into death mode prediction.

In the domain of diffusion model-based protein backbone design and docking, the RFdiffusion tool proposed by Watson et al. successfully applied diffusion models to protein structure generation for the first time, with its designed protein binders exhibiting extremely high affinity and specificity, as experimentally verified [101]. This aids in designing agonists or antagonists targeting specific protein–protein interaction interfaces within cell death pathways. DiffDock, proposed by Gabriele et al., a diffusion model focused on blind docking of small molecules to proteins, is also widely used in academia and industry to accelerate screening of vast compound libraries [102]. Critically, however, we must recognize that the actual improvement in the “hit rate” for virtual screening by these tools is highly dependent on the structural flexibility of the target. For membrane-associated proteins such as GPX4 or the copper-ion-mediated specific oligomerization observed in cuproptosis, the reliability of their predictions still requires extensive calibration through wet-lab experiments.

In molecular representation learning and property prediction, chemical language models based on the Transformer architecture, which capture complex relationships between atoms akin to words in text via “attention mechanisms,” have become potent tools. The ChemBERTa model proposed by Chithrananda et al. and the Molformer tool by Wu et al., pre-trained on massive chemical libraries such as ZINC, can efficiently predict ADMET properties of peptides and compounds [29,103]. This provides researchers with a front-line virtual screening filter, enabling the rapid elimination of lead compounds that inherently possess cardiotoxicity, hepatotoxicity, or poor membrane permeability, which is valuable for molecules such as copper ionophores that require a delicate balance between metal binding and cellular uptake. Furthermore, tools such as Uni-Mol, proposed by Zhou et al., can not only predict molecular properties but also directly predict ligand–protein binding affinities, facilitating the rapid enrichment of potential candidates from libraries containing millions of molecules [104].

However, the most critical and pressing technical bottleneck currently faced by all the above tools is precisely how to specifically predict and distinguish different cell death phenotypes. Currently, the vast majority of tools can only broadly predict “cytotoxicity” or “binding affinity” but cannot directly answer whether a molecule induces ferroptosis or cuproptosis. This necessitates future model architectures that integrate multimodal data. For instance, could predicted protein–ligand complex structures be combined with pathway-level cascade models trained on multi-omics data? A highly promising direction involves jointly training structure predictions from AlphaFold 3 or Chai-1 with supervised signals extracted from specialized databases such as FerrDb and LGod1 to develop a next-generation AI capable of performing “cell death mode classification” directly based on the target interaction pattern. Some work currently utilizes deep learning to dissect non-apoptotic RCD mechanisms; for example, Jin et al. successfully constructed a diagnostic and prognostic model for PAN-related cell death pathways in gastric cancer by integrating CNN, BiLSTM, and cross-attention mechanisms, demonstrating the feasibility of using deep learning to identify non-apoptotic regulated cell death-related molecules from multi-omics data [105]. However, this direction is still nascent and generally lacks in-depth discussion of predictive outcomes versus contradictory experimental findings. This represents the true technological frontier that the field must conquer to achieve the qualitative leap from “virtual screening” to “mechanism prediction.”

## 3. Application of Deep Learning in Drug Discovery for Non-Apoptotic RCD Mechanisms

As the molecular pathways of non-apoptotic RCD mechanisms are gradually elucidated, drug development targeting these pathways has become a frontier hotspot. However, non-apoptotic RCD targets often present challenges such as structural dynamics and complex protein-protein interaction interfaces. With the continuous advancement of deep learning technologies, models capable of understanding molecular structure and predicting bioactivity and drug-likeness have been built. These models enable highly efficient screening of vast chemical libraries, the exploration of natural products, and the rapid prediction of new repurposing opportunities for known drugs, thereby greatly accelerating the process of discovering lead compounds targeting non-apoptotic RCD mechanisms [106].

**Figure 2:**
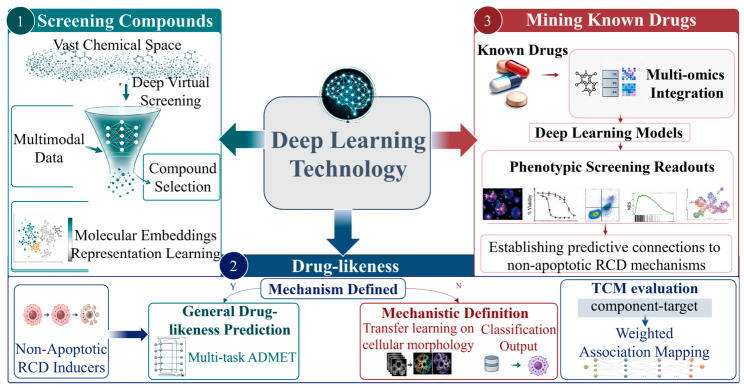
Application of deep learning in drug discovery for non-apoptotic RCD mechanisms. The figure illustrates three major application scenarios of deep learning in non-apoptotic RCD mechanisms and demonstrates the advantages and disadvantages, current status, and future prospects of deep learning in this field.

This section will delineate the three core directions of deep learning applications in this field (see Figure 2). Direct validation evidence must satisfy all three criteria: studies that involve tumor, non-apoptotic RCD mechanisms, and drug discovery. Near-application or transferable frameworks satisfy only a subset of the above criteria; such studies serve as methodological references or are used to extend the discussion. Prospective predictions refer to studies that do not involve non-apoptotic RCD mechanisms, do not involve tumors, or only address general drug discovery; these studies are cited exclusively when discussing methodological potential or future directions. The cited existing studies will be listed in Table 4, summarized and categorized.

### 3.1. Screening for Compounds That Induce Non-Apoptotic RCD Mechanisms

The core challenge in drug discovery targeting non-apoptotic RCD mechanisms lies in rapidly and accurately identifying highly active and selective lead compounds from chemical spaces comprising hundreds of millions of molecules [107]. Deep learning models achieve virtual screening of compound libraries by directly learning complex mapping relationships from molecular structures [108].

In explorations employing deep learning to screen for compounds that induce non-apoptotic RCD mechanisms, significant differences exist between studies regarding the depth of tool validation and the strategies for improving hit rates. These differences directly impact the reliability and generalizability of their methodologies. Yang et al.’s study provides a complete and highly convincing validation loop. They used a supervised Directed Message Passing Neural Network (D-MPNN) to directly learn the anti-hepatocellular carcinoma activity labels of known compounds from the PRISM dataset and subsequently scored over 6 million molecules from the ZINC15 library [109]. The core advantage of this strategy is its effective reduction of false positives and focus on novel scaffolds, achieved through ensemble learning from 20 independent models and secondary filtering based on chemical structural novelty (Tanimoto distance ≥ 0.7). The validation workflow went beyond simple activity re-testing, using orthogonal methods, including ICP-MS, CETSA-WB, and functional proteomics to fully demonstrate and elucidate the novel mechanism of action of the most active candidate molecule, LGOD1, which induces cuproptosis by targeting the copper chaperone protein CCS.

In comparison, Sun et al. used deep learning as a “regression analysis” tool for known active drugs, employing a Graph Convolutional Network (GCN) to predict potential targets of berberine (BBR) [110]. Although the study experimentally confirmed the direct binding of BBR to the top-ranked target Gli1 via Surface Plasmon Resonance and Cellular Thermal Shift Assay, its validation core revolved around confirming an association with the known mechanism of ferroptosis, rather than discovering a completely novel cell death form. The comparative advantage of this method lies in its ability to bypass large-scale focused library screening and directly decipher the complex mechanisms of action of multi-target drugs, such as natural products.

Sun et al. pioneered a fundamentally different technical route. Instead of ligand structure-based or phenotypic screening, they used an AI-driven generative Transformer model to de novo “design” a novel peptide inhibitor, SK56, targeting the GSDMD-NT pore [111]. Their hit rate improvement did not rely on massive libraries but was achieved by the model generating only a tiny set of novel candidate molecules. In terms of validation, the study not only demonstrated high efficacy in vitro and in vivo but also provided an atomic-level explanation of how SK56 blocks the pore, using lipid bilayer nanoparticle experiments, mutation-abolition assays, and cryo-EM docking simulations. The validation depth far exceeded the scope of conventional small-molecule interaction studies. Although this study employs sepsis as a disease model rather than representing a direct case of anti-tumor drug discovery, its strategy of targeting the pyroptosis executioner molecule GSDMD-NT, along with its AI-driven design methodology, offers a directly transferable technical route for the precise modulation of pyroptosis in tumor therapy. It is therefore cited here as a near-application example.

In summary, the comparison of these three methods reveals an evolution from “mass screening of known chemical space” to “rational design overcoming undruggable targets.” Yang et al.’s approach is suited for discovering hit compounds with entirely new scaffolds and mechanisms of action but depends heavily on the availability of high-quality training data. Sun et al.’s strategy provides an efficient target deconvolution pathway for already-known active drugs, triggering the exploration of specific death mechanisms. Meanwhile, studies such as those by Sun et al. demonstrate the transformative potential of AI to create regulatory molecules not found in nature, representing a more disruptive future direction.

### 3.2. Drug-likeness Assessment of Potent Low-Toxicity Anticancer Agents Targeting Non-Apoptotic RCD

Drug-likeness assessment is an indispensable step in the drug discovery process, centered on the systematic evaluation of absorption, distribution, metabolism, excretion, and toxicity (ADMET) properties, along with potency potential [112]. Traditional methods, such as empirical rules exemplified by Lipinski’s Rule of Five, while widely applied for synthetic small molecules, often have limited applicability for structurally complex natural products and non-apoptotic RCD inducers [113]. In recent years, computational methods powered by deep learning, learning from large-scale data, are providing more refined, comprehensive, and interpretable drug-likeness assessments [114].

Multiple deep learning-based drug-likeness prediction frameworks have been proposed for general drug-likeness assessment. Although these studies differ in their validation strategies and hit rate improvement mechanisms and are not distinguished by specific non-apoptotic RCD mechanisms, their methodological frameworks can be adapted for the ADMET evaluation of candidate compounds with well-defined cell death mechanisms. Cai et al. utilized ChemBERTa as a foundation, performing self-supervised pre-training on large-scale unlabeled molecular data and jointly optimizing multiple ADMET endpoints via a multi-task loss function. This approach demonstrated excellent generalization across heterogeneous test sets, including FDA-approved drugs, clinically failed compounds, and ZINC non-drug-like molecules, significantly outperforming traditional methods, such as QED and PrOCTOR [115]. Moving further, Li et al.’s CLaSP shifted from discriminative to generative modeling. By integrating multi-task prediction modules for ADMET, physicochemical properties, and synthetic accessibility and introducing a contrastive learning-guided variational autoencoder, it explicitly separated distributions of drug-like and non-drug-like molecules in the latent space [116]. The CHM-FIEFP model proposed by Pan et al. focuses on tackling the difficult problem of identifying the core active components within TCM formulae [117]. This method integrates component–target associations predicted by network pharmacology with disease differentially expressed gene data, employing an entropy weight method to quantify the “fit” of each component to the overall formula efficacy at both the pathway and gene levels, ultimately producing a sortable, interpretable component efficacy list. Although this study did not designate a specific non-apoptotic RCD mechanism as the prediction target, its multimodal fusion strategy and interpretability design provide valuable methodological insights for research in this direction.

For the specialized assessment of drugs targeting non-apoptotic RCD mechanisms, unlike general drug-likeness evaluation, the evaluation process requires exploring specific mechanistic validation dimensions, because these forms of cell death each possess unique molecular markers and morphological characteristics. A clear decision logic exists here: for compounds already confirmed to induce a specific non-apoptotic regulated death mechanism, the focus of subsequent development is general drug-likeness prediction; for compounds with an unclear mechanism of action, the priority is first to investigate which specific non-apoptotic RCD mechanism they induce. Defining the mechanism is a prerequisite for drug-likeness prediction. Stachura et al. applied Deep Transfer Learning (DTL) to analyze bright-field microscopy images, accurately distinguishing ferroptosis from apoptosis, necroptosis, and other death modes through cellular morphological features [118]. A direct application of this technical strategy led to the discovery that the PLK1 inhibitor volasertib acts as a ferroptosis inducer. Subsequent experiments in B-ALL cell lines and patient samples confirmed that volasertib treatment upregulated the expression of ferroptosis-related genes, and this effect could be specifically reversed by ferroptosis inhibitors, thus completing a closed loop from computational prediction to experimental validation.

### 3.3. Mining the Non-Apoptotic Regulated Cell Death-Inducing Effects of Known Drugs

Drug repurposing is highly favored for its low development risk and shortened timelines [119]. In recent years, deep learning, by integrating multi-omics data and phenotypic screening data, has enabled the prediction of connections between known drugs and non-apoptotic RCD pathways [120]. Currently, much of this field remains at a speculative stage.

In multi-layered biological network representation learning, the high density of gene nodes often causes random walk strategies to produce embedding bias, where topological features associated with genes dominate the representation results. The DREAMwalk framework developed by Bang et al. effectively mitigates this problem in drug–gene–disease multi-layered heterogeneous networks through a semantic-guided random walk strategy [121]. Compared to traditional heterogeneous network models, this method showed superior ranking performance in recovering known drug–disease associations, with its core benefit being improved priority ranking accuracy of potential associations, rather than directly increasing the overall hit rate. This strategy can be applied to construct knowledge graphs containing key nodes for ferroptosis and necroptosis, such as GPX4, ACSL4, and RIPK1, to mine potential links between drugs and specific death mechanisms in a high-dimensional semantic space. However, the association conclusions currently drawn by such models remain statistical inferences and have not been systematically compared against mechanistic evidence from perturbation transcriptomics or loss-of-function experiments. Their effectiveness as tools for experimental priority ranking has been validated in some cases, but their reliability as standalone discovery tools remains insufficient.

At the functional evaluation level, the “single-cell array-enhanced AI image recognition system” proposed by Li et al. advances drug screening to a higher resolution [122]. Unlike traditional population-based cell viability endpoint assays, this system achieves precise single-cell arrangement through micromachining technology and, combined with a CNN trained using multi-class cross-entropy loss, can not only discriminate live/dead status but also perform fine-grained classification of damage extent. A direct comparative advantage of this method lies in its ability to resolve phenotypic heterogeneity masked by conventional endpoint methods, thereby improving the detection sensitivity and quantification accuracy for anticancer drug-induced cell death effects. Although this study does not currently distinguish between specific cell death modalities, its fine-grained damage grading capability holds potential discriminative value for differentiating death modes accompanied by pronounced morphological differences, such as pyroptosis and necroptosis. Moreover, its single-cell resolution may help reveal phenotypic heterogeneity that is otherwise masked by conventional endpoint assays. It is therefore cited here as a near-application validation.

In drug–disease association prediction, deep learning models based on heterogeneous networks have achieved reproducible predictive performance. For example, the SpHN-VDA model proposed by Ren et al., by constructing a drug–virus heterogeneous network and incorporating multi-modal semantic information, successfully realized antiviral drug repurposing [123]. This model was systematically validated on public datasets, outperforming multiple baseline methods, indicating that multi-layered network representation learning can effectively capture drug-target associations. However, directly transferring such models to cell death research still faces critical gaps: existing evaluations largely remain at the level of binary association prediction and have not been experimentally verified in real scenarios differentiating between multiple cell death modes. Although this study focuses on antiviral drug repurposing and has no direct relevance to the cell death theme, its spatial hierarchical network construction method can be transferred to the construction of multi-layer “drug–gene–signaling pathway–cell death type” networks. It is therefore discussed here as a methodological analogy.

In summary, from network-level association prediction to single-cell resolution phenotypic validation, these studies constitute a multi-scale path for drug repurposing and the mining of non-apoptotic RCD mechanisms. Single-cell phenotypic validation has shown clear advantages in improving detection resolution, while heterogeneous network models typically contribute to enhancing priority ranking efficiency in association prediction, rather than directly increasing the hit rate. The critical future challenge lies in systematically integrating these multi-level methods and experimentally confirming the new mechanisms revealed computationally through prospective validation.

**Table 4:** Representative deep learning studies for drug discovery targeting non-apoptotic RCD mechanisms. Studies are categorized by task type, targeted RCD mode, model architecture, dataset size, key finding, and principal limitation. Detailed contents of the table are presented in Supplementary Table S2.

Task Type	Targeting Type	Model	Dataset Size	Core Finding	Key Limitation
Lead compound screening [109]	Cuproptosis	D-MPNN ensemble	~900 (training) > 6 million (screening)	Discovery of LGOD1, targeting CCS to induce cuproptosis	Dependent on high-quality training data
Drug target prediction [110]	Ferroptosis	GCN	Single drug	BBR targeting Gli1 associated with ferroptosis	Correlated with known mechanisms, not a novel discovery
Peptide-based inhibitor design [111]	Pyroptosis	Generative Transformer	<10 candidate peptides	Designed SK56 to block GSDMD-NT pore	Peptide-specific; small-molecule generalizability remains to be validated
Drug-likeness prediction [115]	Nondiscriminatory	ChemBERTa and multi-task learning	>1 million molecules	Universal high-precision ADMET scoring	Not specifically optimized for RCD
Drug-likeness optimization [116]	Nondiscriminatory	CLaSP contrastive learning VAE	Not disclosed	Interpretable drug similarity scoring	High computational cost
Active ingredient identification in compound formulas [117]	Nondiscriminatory	CHM-FIEF and entropy weight method	Not disclosed	Identification and ranking of core active ingredients in compound formulas	Dependent on network pharmacology accuracy
Cell death mode classification [118]	Ferroptosis	Deep transfer learning	Not disclosed	Identified volasertib as a ferroptosis inducer	Dependent on morphological annotations
Drug–disease repurposing * [121]	Extendable	DREAMwalk semantic random walk	Not disclosed	Improved association ranking precision	Conclusions are based on statistical inference
Single-cell phenotypic screening * [122]	Nondiscriminatory	CNN with multi-class cross-entropy	Not disclosed	Enhanced damage detection resolution	Does not differentiate between cell death modes
Drug–virus repurposing * [123]	Extendable	SpHN-VDA	Not disclosed	Multi-level network representation learning captures drug–target associations	Lack of experimental validation across multiple cell death modes

* Transferable frameworks not yet validated in the context of non-apoptotic RCD mechanisms.

## 4. Conclusions and Future Perspectives

Despite the unprecedented acceleration that deep learning has brought to drug discovery targeting non-apoptotic RCD mechanisms, the field now stands at a critical crossroads. Through a structured narrative review of the full technical pipeline—from molecular representation and multimodal fusion to compound screening and drug-likeness assessment—this review delineates the progress of deep learning in this domain. Research directly aimed at discovering antitumor agents that target specific non-apoptotic RCD mechanisms remains relatively sparse. Although a substantial body of work on general drug-likeness prediction, cell death regulation in non-cancer contexts, and screening methods that do not distinguish among death modalities offers valuable technical references, these approaches still require targeted development toward non-apoptotic RCD mechanisms. More importantly, if these computational successes are to be translated into clinical candidates or gain regulatory endorsement, a fundamental issue must be confronted: a vast gap remains between AI predictions and biological validation as well as industrial standards. The essence of this gap lies not in the fine-tuning of model architectures, but in the absence of discriminatory capability and trust mechanisms. Current models lack sufficient specificity in distinguishing among multiple cell death modalities, making it difficult to confidently identify the screened molecules as genuine mechanism-specific inducers. Furthermore, the absence of standardized external blind-testing systems and interpretability guarantees has left the trust of industry and regulatory agencies in AI predictions confined to the level of isolated case demonstrations. The application of deep learning has moved from the stage of “whether it can predict” to “whether it can discriminate,” and it is precisely this lack of discriminatory capability that constitutes the root cause of the chasm hindering the translation from computational prediction to industrial application.

1. Addressing reliability and mechanistic misattribution in discriminative screening

Current virtual screening tools largely predict cytotoxicity or target affinity in a generalized manner, yet what the pharmaceutical industry truly needs is discriminative screening technology capable of precisely distinguishing among cell death modalities. For medicinal chemists, a compound that simply kills cells is relatively easy to obtain; a compound that specifically induces ferroptosis without triggering apoptosis or necroptosis, however, holds potential value for circumventing classical drug-resistance pathways. Existing models are prone to mechanistic misattribution in this task—what they learn may primarily reflect statistical correlations between specific chemical scaffolds and loss of cell viability, rather than genuine programmed cell death induction mechanisms. This results in low translational efficiency from screening to wet-lab validation. In the future, explainable AI should move beyond merely outputting attribution heatmaps and evolve into systems capable of tracing and verifying the decisive physicochemical logic between molecules and key execution proteins (such as GPX4, FDX1, MLKL, and GSDMD), thereby providing testable mechanistic evidence for predictions. Establishing this capability will directly influence whether such methods gain broad acceptance within the pharmaceutical community.

2. Generalization challenges under data scarcity and regulatory science foresight

For mechanisms such as cuproptosis, the number of known active molecules is extremely limited, making models under few-shot learning conditions prone to overfitting spurious correlations in the data, with a tendency to rely on scaffold-based features rather than the intrinsic nature of pharmacophores. Future efforts should focus on constructing multimodal databases dedicated to non-apoptotic RCD mechanisms, encompassing high-content morphological profiles, dynamic protein–metabolite interactomes, chemical perturbation structure–transcriptional response matrices, and standardized annotations including negative labels and failed experimental data. Furthermore, from a long-term regulatory science perspective, when entirely novel virtual compounds predicted by AI enter preclinical evaluation, regulatory agencies will face a new assessment challenge: how to evaluate the long-term safety of a molecule designed de novo that lacks an evolutionary lineage traceable to natural products or known drugs. It is therefore necessary for the field to begin developing, from this point forward, a standardized validation framework for AI-assisted compound discovery. This should include unified external blind-test benchmarks (rather than relying solely on holdout-set AUC), mandatory rescue experiments targeting key pathways as verification mechanisms, and reasonable quantification of predictive uncertainty. Without such standards, confidence from both industry and regulatory agencies in AI-discovered molecules is unlikely to advance beyond isolated case demonstrations.

3. From static structure prediction toward dynamic biological context simulation

Another noteworthy technical bottleneck is the spatiotemporal adaptability of models. Neither static structures predicted by AlphaFold nor phenotypic classifications based on single-frame images can adequately capture the kinetic tipping points of uncontrolled lipid peroxidation during ferroptosis, or the nonlinear transition from normal metabolism to the copper lethality threshold in cuproptosis. Future models must progress from static structure–activity relationships toward dynamic, context-aware prediction systems. This means models should integrate real-time information on the metabolic reprogramming state of tumors, redox potential, and compensatory defense networks. Developing technologies capable of predicting cell death modality biases based on cellular context will represent an important breakthrough point for advancing precision medicine applications.

**Figure 3:**
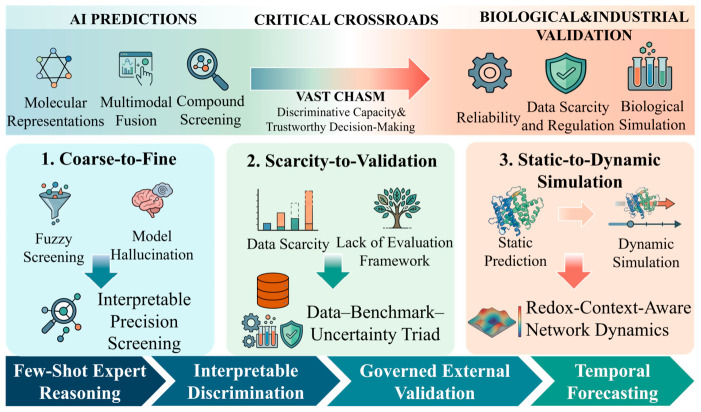
Current challenges and future perspectives. The figure summarizes the current status and pain points that need to be addressed in the field of deep learning in non-apoptotic RCD mechanisms and highlights future challenges and technological frontiers.

In summary, this review proposes that what the field most urgently requires is not yet another more complex general-purpose molecular encoder, but rather expert-level few-shot reasoning capabilities and mechanistically interpretable discriminative power tailored to non-apoptotic RCD mechanisms. Standardized data governance, a rigorous culture of external closed-loop validation, and temporally resolved predictive models capable of discerning which fate a cell ultimately adopts amid converging death signals are the key factors that will propel this direction from literature-based studies toward clinical application. Breakthroughs at these technical frontiers will determine whether deep learning can substantively reshape the industrial discovery paradigm for cell-death-targeting antitumor drugs and, on that basis, gradually earn the recognition of regulatory science (see Figure 3).

## Figures and Tables

**Table 3:** Overview of core deep learning architectures for studying non-apoptotic RCD mechanisms.

Deep Learning Architecture	Typical Application Scenarios	Maturity and Prospects
Graph Neural Networks (GNNs)	Predicting the probability of compounds inducing specific activities like ferroptosis and cuproptosis; identifying key targets.	Relatively mature for ferroptosis scenarios; highly prone to overfitting in small-sample scenarios like cuproptosis, requiring cautious validation.
Convolutional Neural Networks (CNNs)	Analyzing high-content cellular images to classify cell death modalities via morphological changes.	Under development; can serve as an orthogonal validation tool, but reliability as a stand-alone predictive method is limited by annotation granularity.
Recurrent Neural Networks/Transformers	Generating novel molecular sequences with bioactivity; predicting protein-protein interactions.	Existing cell viability data labels are mixed, making it difficult to provide specific reward/punishment signals for pyroptosis or necroptosis.
Autoencoders/Variational Autoencoders (VAEs)	Learning low-dimensional representations of chemical space to generate novel molecules with specific properties and build virtual libraries.	As the technical foundation for generative models, its capacity to explore new mechanisms depends on the chemical diversity of the starting library.
Multimodal Deep Learning Networks	Integrating multi-source data (e.g., molecular structures, gene expression, pathology images) for systems pharmacology prediction.	At the proof-of-concept stage, the major bottleneck is the extreme scarcity of high-quality, paired multimodal datasets.
Graph Attention Networks (GATs)	Analyzing signaling pathways or protein-protein interaction networks to identify key targets regulating cell death.	Relatively mature as an auxiliary tool for target discovery and hypothesis generation; predicting compound activity remains an indirect inference.

## Data Availability

No new data were created or analyzed in this study. Data sharing is not applicable to this article.

## Supplementary Materials

The following supporting information can be downloaded at: https://www.mdpi.com/article/10.3390/ph19060851/s1, Table S1: Benchmark of key data resources for research on non-apoptotic regulated cell death mechanisms; Table S2: Complete summary of deep learning applications in drug discovery related to non-apoptotic regulated cell death mechanisms.

## Abbreviations

The following abbreviations are used in this manuscript:

ACSL4	Acyl-CoA Synthetase Long Chain Family Member 4
ADMET	Absorption, Distribution, Metabolism, Excretion, and Toxicity
AI	Artificial Intelligence
AUC	Area Under the Curve
B-ALL	B-cell Acute Lymphoblastic Leukemia
CCLE	Cancer Cell Line Encyclopedia
CETSA	Cellular Thermal Shift Assay
CMap	Connectivity Map
CNN	Convolutional Neural Network
cryo-EM	Cryo-Electron Microscopy
CTRP	Cancer Therapeutics Response Portal
D-MPNN	Directed Message Passing Neural Network
DLAT	Dihydrolipoamide S-Acetyltransferase
DTL	Deep Transfer Learning
FDX1	Ferredoxin 1
GAT	Graph Attention Network
GDSC	Genomics of Drug Sensitivity in Cancer
GNN	Graph Neural Network
GPX4	Glutathione Peroxidase 4
GSDMD	Gasdermin D
GSDMD-NT	Gasdermin D N-Terminal domain
GSH	Glutathione
GTN	Gated Transformer Network
ICP-MS	Inductively Coupled Plasma Mass Spectrometry
IL-1β	Interleukin 1 Beta
LDH	Lactate Dehydrogenase
LIAS	Lipoic Acid Synthetase
LPCAT3	Lysophosphatidylcholine Acyltransferase 3
MDA	Malondialdehyde
MLKL	Mixed Lineage Kinase Domain Like Pseudokinase
OOD	Out-of-Distribution
PI	Propidium Iodide
pMLKL	Phosphorylated MLKL
PPI	Protein–Protein Interaction
PRISM	Profiling Relative Inhibition Simultaneously in Mixtures
QSAR	Quantitative Structure-Activity Relationship
RCD	Regulated Cell Death
RIPK	Receptor Interacting Serine/Threonine Kinase
RNN	Recurrent Neural Network
ROS	Reactive Oxygen Species
SELFIES	Self-Referencing Embedded Strings
SMILES	Simplified Molecular Input Line Entry System
SPR	Surface Plasmon Resonance
TCM	Traditional Chinese Medicine
UMAP	Uniform Manifold Approximation and Projection
VAE	Variational Autoencoder
XAI	Explainable Artificial Intelligence
