# Supplementary material for: Deep Learning for Anticancer Drug Discovery Targeting Non-Apoptotic Regulated Cell Death Mechanisms
Source: Pharmaceuticals (Basel). 2026 May 29;19(6):851. doi: 10.3390/ph19060851 (PMC13304829; doi:10.3390/ph19060851)
Supplement: Supplementary file 1 [file pharmaceuticals-19-00851-s001.zip › pharmaceuticals-4284922-supplementary.pdf]

Supplementary Table S1. Benchmark of key data resources for research on non-apoptotic regulated cell death mechanisms. The table summarizes publicly available datasets spanning general compound libraries, multi-omics and drug perturbation resources, drug–target association databases, protein structure repositories, phenotypic imaging and knowledge graph platforms, and mechanism-specific databases. For each resource, we report its data modality, approximate scale, cell-line or tissue coverage, endpoint readout, specificity for non-apoptotic regulated cell death mechanisms, reliability of positive and negative labels, suitable prediction tasks, recommended data-splitting strategy, principal limitations, and annotation content. Resources are grouped into seven categories to reflect their primary utility in the drug discovery pipeline.

Resource Category	Resource Name	Modality	Approximate Scale	Cell Line/Tissue Coverage	Endpoint	Death Mode Specificity	Positive/Negative Label Reliability	Suitable Tasks	Recommended Split Strategy	Main Limitations	Annotation Content
**General Compound Libraries**	PubChem	Compound structure + bioactivity	~110,000,000 compounds, ~300,000 bioassays	Multi-type assays	Multiple endpoints	Partial	Moderate (high data heterogeneity)	Virtual screening, pre-training data source	Chemical space/assay type split	High data noise; death mode annotations scarce and unstructured	Active/Inactive only
	ChEMBL	Compound structure + target activity	~2,400,000 activity records	Multi-species, multi-target	IC_{50}, Kd, Ki, etc.	Indirect	High (strong data heterogeneity)	Target binding prediction, QSAR modeling	Compound scaffold/target family split	Endpoint is target binding, not death phenotype	Compound-target-activity value
	ZINC	Compound 3D structure	~230,000,000 purchasable compounds	—	Virtual structure	None	Not applicable	Virtual screening library, molecular generation training	Chemical space/scaffold split	Pure chemical library; requires integration with activity prediction models	Purchasability, 3D conformation
**Multi-omics & Drug Perturbation Libraries**	PRISM,	Compound structure + cell viability	~4,500 compounds, ~900 cell lines	Pan-cancer, 26 tissue types	Cell viability (ATP quantification)	None	Moderate (live/dead binary labels only)	Lead compound screening, sensitivity/resistance prediction	Cell-line-level split	Lacks death subtype information	Cell viability value
	DepMap/CCLE	Genomics + CRISPR screening	~1,800 cell lines, ~18,000 genes	Pan-cancer, 30+ tissue types	Gene dependency, expression, mutation	Indirect	High (rigorous quality control)	Target discovery, contextual feature construction	Chromosome arm/tissue-level split	No direct compound activity data	Gene dependency score, expression level
	GDSC	Compound structure + drug sensitivity data	~518 compounds, ~1,000 cell lines	Pan-cancer	IC_{50}, AUC	None	Moderate (batch effects common)	Drug sensitivity prediction	Cell-line/tissue-level split	Limited compound coverage	Gene/pathway/IC_{50}
	CTRP	Compound structure + drug sensitivity data	~860 compounds, ~900 cell lines	Pan-cancer	AUC	None	Moderate (limited concordance with GDSC)	Drug sensitivity prediction, repurposing	Cell-line/tissue-level split	Batch effects require attention when integrating with GDSC	Gene/pathway/AUC
	CMap/LINCS	Transcriptomic perturbation profiles	~20,000 compounds, L1000 covering ~1,300 genes	Multiple tumor and non-tumor cell lines	Gene expression perturbation signature	Indirect	Moderate (L1000 has higher noise)	Mechanism-of-action inference, drug repurposing	Cell-line/compound scaffold split	Endpoint is expression perturbation, not death phenotype	Perturbation gene signature
**Phenotypic Images & Knowledge Graphs**	Cell Painting/ IDR	Drug-target-indication association	~4,900 drugs, ~5,000 targets	—	Target, pathway, indication	Indirect	High (approved drug information)	Drug repurposing, target-death pathway association inference	Drug/target/indication hierarchical split	Non-specialized death information	Drug-target-indication-mechanism association
	KEGG	Protein-ligand binding data	~2,000,000 binding records	Multi-species, multi-target	Kd, IC_{50}, Ki, etc.	Indirect	High (detailed experimental records)	Molecular docking benchmark, binding affinity prediction	Target/compound scaffold/sequence similarity split	Endpoint is binding, not functional effect	Target-ligand-affinity-experimental conditions
	Reactome	Protein 3D structure	~200,000 structures	Multi-species	3D coordinates	Potential	Extremely high (X-ray/NMR/cryo-EM)	Structure prediction, molecular docking, protein design	Sequence homology/protein family split	Static structures cannot reflect death dynamics	3D coordinates-active site-metal coordination-modifications
**Drug-Target Association Databases**	DrugBank	High-content morphological images	~30,000 compounds	U2OS and various cell lines	Multi-channel fluorescence images	Potential	Low-Moderate (requires manual annotation)	Phenotypic classification, preliminary death mode discrimination	Biological replicate/batch split	Death mode annotations extremely scarce	Relatively comprehensive but limited coverage
	BindingDB	Pathway/network	~540 pathways	Applicable to multiple species	Pathway maps, molecular interactions	Indirect	High (literature-curated)	Knowledge graph construction, mechanistic association inference	Pathway/functional-level split	Static pathways, lacks cellular context	Relatively comprehensive but limited coverage

**Protein Structure Database**	PDB	Pathway/network	~2,000+ reactions	Applicable to multiple species	Pathway maps, molecular interactions	Indirect	High (literature-curated)	Knowledge graph construction, mechanistic association inference	Pathway/functional-level split	Static pathways, lacks cellular context	Relatively comprehensive but limited coverage
**Specialized Disease Databases**	FerrDb	Experimentally validated ferroptosis regulators	~1,000+ genes/molecules	Multi-species	Inducer/inhibitor/marker labels	High (specifically annotated for ferroptosis)	Extremely high (manual literature curation)	Ferroptosis-specific training set, target validation	Literature/functional category split	Covers only ferroptosis	Molecule-death type-target association

Supplementary Table S2. Complete summary of deep learning applications in drug discovery related to non-apoptotic regulated cell death mechanisms. The table summarizes all studies cited in Section 3, categorizing them by task type, disease and cell death mechanism, data modality, dataset size, model type, experimental validation approach, key finding, and limitation or transferability. For rows where dataset size is unavailable or not applicable, this is explicitly stated. Rows describing studies not specific to a particular RCD type are retained where the methodological framework is transferable to non-apoptotic RCD research.

Task	Disease / RCD type	Data type	Dataset size	Model	Experimental validation	Key finding	Limitation / Transferability
**Lead compound screening**	HCC; Cuproptosis	Compound structures (PRISM; ZINC15)	PRISM: ~900 compounds ZINC15: >6M compounds	D-MPNN (20-model ensemble)	ICP-MS, CETSA-WB, functional proteomics	LGOD1 identified as a novel cuproptosis inducer targeting CCS	Highly dependent on training data quality; applicability to other RCD types requires further validation
**Drug target prediction / MoA**	Colorectal cancer; Ferroptosis	Known drug structure (berberine, BBR)	Not applicable (single-drug study)	GCN	SPR, CETSA, gene / lipid peroxidation assays	BBR targets Gli1 and associates with ferroptosis and energy metabolism disruption	Mechanism validation is associative; does not discover novel death modes
**Peptide inhibitor design**	Sepsis / inflammation (non-cancer); Pyroptosis	GSDMD-NT pore structure	<10 candidate peptides	AI-driven generative Transformer	Lipid bilayer assay, cryo-EM docking, in vitro/in vivo activity	SK56 identified as a novel GSDMD pore blocker; atomic-level mechanism explained	Peptide-focused study; generalizability to small molecules is not yet established
**Drug-likeness prediction**	Pan-cancer / non-cancer (not RCD-specific)	Unlabeled molecular structures (ZINC non-drug set, etc.)	>1M molecules (pretraining); multiple benchmark sets	ChemBERTa + multi-task learning	Comparison with QED, PrOCTOR, etc.	High-accuracy general ADMET scoring; strong generalizability	Not specifically optimized for non-apoptotic RCD compounds
**Drug-likeness optimization**	Pan-cancer (WEE1) (not RCD-specific)	Molecular structures & properties	Not explicitly disclosed (multi-source training data)	CLaSP: contrastive VAE + multi-task prediction	Retrospective linkage to CYP inhibition, neurotoxicity, etc.	Interpretable drug-likeness score; correlates with clinical toxicity	High computational cost; requires large-scale pretraining
**Herbal formula active ingredient identification**	Gastric cancer (not RCD-specific)	Network pharmacology; differential gene expression	Not explicitly disclosed (formula-pertinent data)	CHM-FIEFP + entropy weight method	Experimental validation of ferulic acid as formula surrogate	Objective identification and ranking of active components in herbal formulas	Relies on accuracy of network pharmacology predictions; needs experimental confirmation
**Death mode classification / new inducer discovery**	B-ALL; Ferroptosis	Bright-field microscopy images (cell morphology)	Not explicitly disclosed (multiple-death-mode image set)	Deep transfer learning (DTL)	Gene expression; rescue by specific inhibitors	Volasertib discovered as ferroptosis inducer; discrimination among death modes	Dependent on high-quality morphological annotation; primarily tested in adherent cells
**Drug–virus repurposing***	Non-cancer (framework study)	Drug–virus heterogeneous network	10-fold CV on public datasets (exact size not disclosed)	SpHN-VDA	Performance comparison with multiple baselines	Multi-level network representation captures drug–target associations	Not yet adapted to cell death context; lacks multi-mode death experimental validation
**Drug–disease association***	Multi-disease (extendable to ferroptosis, etc.)	Drug–gene–disease multilayer network	Not explicitly disclosed (heterogeneous network)	DREAMwalk: semantic-guided random walk	Recovery of known associations; ranking performance evaluation	Improved ranking of potential drug–disease associations	Conclusions are statistical; lacking mechanistic experimental validation
**Single-cell phenotypic screening / efficacy evaluation***	Anticancer drug screening (not RCD-specific)	Single-cell array images	Not explicitly disclosed (image-based screening assay)	CNN (multi-class cross-entropy loss)	Graded damage scoring in single-cell assays	Enhanced detection resolution for drug-induced cell damage	Does not distinguish among specific RCD types; potential for pyroptosis/necroptosis discrimination noted

* Transferable frameworks not yet validated in the context of non-apoptotic regulated cell death mechanisms.
